# Establishment and characterization of an immortalized epithelial cell line from human gallbladder

**DOI:** 10.3389/fonc.2022.994087

**Published:** 2022-10-28

**Authors:** Ziyi Wang, Shijia Wang, Ziheng Jia, Yuhao Zhao, Mao Yang, Weikang Yan, Tao Chen, Dongxi Xiang, Rong Shao, Yingbin Liu

**Affiliations:** ^1^ Department of Biliary-Pancreatic Surgery, Renji Hospital Affiliated to Shanghai Jiao Tong University School of Medicine, Shanghai, China; ^2^ Shanghai Cancer Institute, State Key Laboratory of Oncogenes and Related Genes, Shanghai, China; ^3^ Shanghai Key Laboratory of Biliary Tract Disease, Renji Hospital, Shanghai, China; ^4^ Shanghai Research Center of Biliary Tract Disease, Renji Hospital, Shanghai, China; ^5^ Department of Pharmacology and Biochemistry, Shanghai Jiao Tong University School of Medicine, Shanghai, China

**Keywords:** gallbladder, gallbladder cancer, epithelium cell, cell lines, immortalization

## Abstract

**Background:**

Although a plethora of studies have employed multiple gallbladder cancer (GBC) cell lines, it is surprisingly noted that there is still lack of a normal gallbladder epithelial cell line as a normal counterpart, thus impeding substantially the progress of mechanistic studies on the transformation of normal epithelial cells to cancer. Here, we created a normal gallbladder epithelial cell line named L-2F7 from human gallbladder tissue.

**Methods:**

Gallbladder tissues from a diagnosed cholecystitis female patient were collected, and epithelial cells were enriched by magnetic cell sorting. Then, the cells were immortalized by co-introduction of human telomerase reverse transcriptase (hTERT) and Simian virus 40 large T antigen (LT-SV40) *via* a lentivirus infection system. After clonal selection and isolation, L-2F7 cells were tested for epithelial markers CK7, CK19, CK20, and CD326, genomic feature, cell proliferation, and migration using Western blot, immunofluorescence, whole genome sequencing, karyotyping, and RNA sequencing. L-2F7 cells were also transplanted to Nude (nu/nu) mice to determine tumorigenicity.

**Results:**

We successfully identified one single-cell clone named L-2F7 which highly expressed epithelial markers CD326, CK7, CK19, and CK20. This cell line proliferated with a doubling time of 23 h and the epithelial morphology sustained over 30 passages following immortalization. Transient gene transduction of L-2F7 cells led to expression of exogenous GFP and FLAG protein. L-2F7 cells exhibited both distinct non-synonymous mutations from those of gallbladder cancer tissues and differential non-cancerous gene expression patterns similar to normal tissue. Although they displayed unexpected mobility, L-2F7 cells still lacked the ability to develop tumors.

**Conclusion:**

We developed a non-cancerous gallbladder epithelial cell line, offering a valuable system for the study of gallbladder cancer and other gallbladder-related disorders.

## Introduction

The gallbladder, a hollow pear-shaped sac, is anatomically located on the visceral surface of the right lobe of the liver and connected with the liver and duodenum by biliary tracts (1). The primary function of the gallbladder is to store bile synthesized from the liver and release bile in response to cholecystokinin, when intake food moves into the upper digestive system (2, 3). Thus, the gallbladder is classified as a digestive organ helping food digestion, particular for fat acid metabolism. The gallbladder wall anatomically consists of three layers: mucosa, muscularis, and serosa (4, 5). The mucosa layer harbors multiple epithelial glands where epithelial cells are the primary site for pathophysiological lesions such as inflammation, stone development, and carcinogenesis (6).

Common gallbladder-related diseases include acute or chronic cholecystitis, cholecystolithiasis, gallbladder polyps, and gallbladder carcinoma (GBC). GBC is the most common biliary tract malignancy with only 5% of 5-year survival rate and ranked the sixth among gastrointestinal cancers (7, 8). The primary GBC develops from gallbladder epithelial cells that undergo transformation into highly invasive tumor cells (9). Given the availability of primary tumors removed from surgery (10), a number of cell lines of GBC have been established including GBC-SD, NOZ, ZJU-0430, and OCUG1 cells (11). However, it is unexpectedly noted that there is still lack of a non-cancerous normal gallbladder epithelial cell line. The main reason for this shortage is probably owing to rapid senescence of isolated epithelial cells following cell passages in culture, thus emerging as an evident challenge in the genetic and functional characterization between normal epithelial cells and tumor cells. Therefore, the establishment of a normal gallbladder cell line is of paramount importance in the study on malignant transformation of normal epithelial cells to cancer cells. Here, we immortalized and characterized a normal gallbladder epithelial cell line derived from gallbladder tissues of a 30-year-old female patient with cholecystitis *via* introduction of SV-40 large T and HTERT genes. A single-cell clone named L-2F7 was cultured through limiting dilution assay, and its epithelial origin was confirmed by examining epithelial cell markers CK19, CK7, CK20, and CD326, and epithelial cell function. Our data suggest that L-2F7 cells can serve as a normal gallbladder epithelial cell line characteristic of the normal epithelium of the gallbladder.

## Result

### The establishment of the L-2F7 cell line

**Figure 1:**
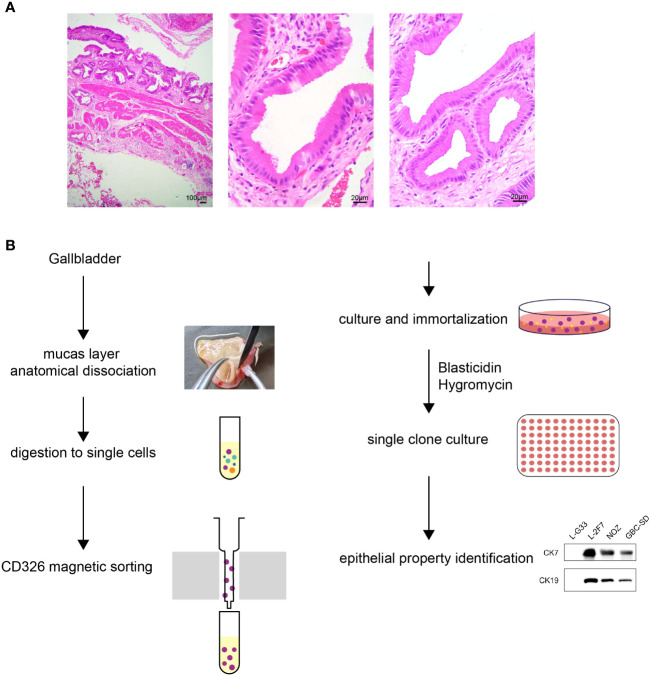
Pathology of gallbladder tissue and the scheme of L-2F7 cell line establishment. Partial fragments of original gallbladder tissues were used for isolating epithelial cells in HE staining, which showed no significant pathological abnormality of gallbladder **(A)**. Gallbladders were collected after the surgical operation and its mucosa layers were then dissociated anatomically. After being digested into single cells, CD326-positive cells were collected using magnetic cell sorting. The primary epithelial cells were plated to culture dishes, immortalized, and cultured in the presence of 200 μg/ml blasticidin and 10 μg/ml hygromycin. Limited dilution assays were employed to select clones for the following further characterization **(B)**.

With regard to the selection of surgically resected gallbladder tissue, the clinical criterion for enrolled gallbladder diseases was set up: 1) no significant epithelium thickness; 2) no evident enlargement or atrophy of gallbladder; 3) no polyp of gallbladder; 4) tissue pathology showed neither severe significant infiltrating inflammation cells nor any abnormal histological structure including atypical hyperplasia. The gallbladder of a 30-year-old female patient diagnosed with cholecystitis met all the above requirements, and the pathological state of this gallbladder was evaluated through HE staining (**Figure 1A**). To isolate and purify epithelial cells from all miscellaneous cells of gallbladder tissue, we discreetly dissected the top lumenal layer from the beneath muscular layer, and subsequently we employed a CD326-positive magnetic cell sorting assay to enrich the epithelial cell population (**Figure 1B**). The cells were then cultured to adhere on culture dishes and immortalized by lentivirus infection with viral vectors carrying the SV40-large T antigen (LT-SV40) and hTERT gene. Following a limiting dilution assay, the cells were dispersed and individually grown in 96-single wells. A single-cell clone named L-2F7 expressing LT-SV40 and hTERT (**Supplementary Figure 1A**), TP53, and Rb (**Supplementary Figure 1B**) was selected for the following characterization of epithelial cells.

### Identification of the epithelial cell property

**Figure 2:**
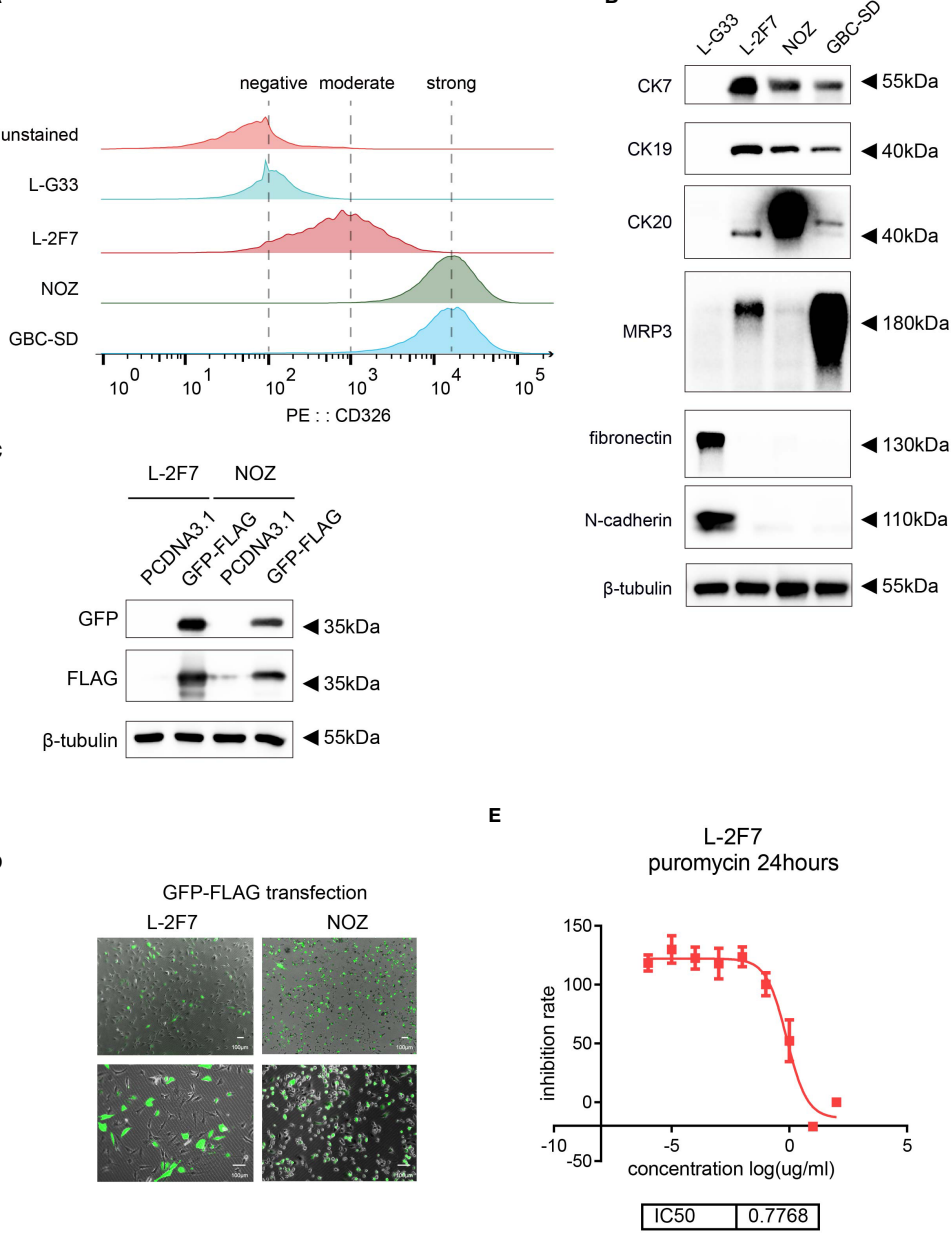
Identification of single clone cells L-2F7 for the epithelial signature. Epithelial markers were detected in L-2F7 cells, gallbladder cancer cell lines NOZ and GBC-SD, and L-G33 cells as a fibroblast control with flow cytometry **(A)**. CK7, CK19, MRP3, fibronectin, and N-cadherin were determined with Western blot **(B)**. L-2F7 cells were transfected by GFP-FLAG, then the expression of GFP and FLAG was detected by Western blot **(C)** and fluorescence was applied for observing the transfection efficiency **(D)**. The sensitivity of puromycin in L-2F7 cells was measured by calculating IC50 from the fitted curve **(E)**.

To validate that L-2F7 cells are epithelial cells, we performed the flow cytometry assay using an anti-CD326 antibody and we found that compared with a gallbladder fibroblast cell line L-G33 as a negative control that was established from another patient, L-2F7 cells expressed CD326, although its level was lower than NOZ and GBC-SD cells (**Figure 2A**). Immunoblotting showed that L-2F7 cells also strongly expressed epithelial cell markers CK7, CK19, and CK20 (**Figure 2B**), consistent with immunofluorescence analysis (**Supplementary Figure 1C**). The strong expression of epithelial markers was maintained during 30 cell passages (**Supplementary Figure 1C**). To exclude the possibility of fibroblast contamination, the most common problem present in primary cell culturing, we determined the expression of fibronectin and N-cadherin by Western blot (**Figure 2B**) and immunofluorescence (**Supplementary Figure 1D**). In sharp contrast with L-G33 cells, L-2F7 cells did not express fibronectin and N-cadherin, supporting that L-2F7 is derived from epithelial cells. As expected, L-2F7 highly expressed MRP3, a well-acknowledged bile acid receptor mostly expressed by gallbladder epithelial cells (**Figure 2B**), implying the possible reservation of normal gallbladder function (12, 13).

To interrogate if this cell line can be used as a common control system to accept exogenous genetic introduction, we transduced a vector carrying the GFP-FLAG gene into L-2F7 cells with lipofectamine transfection. Efficiency of the transient transfection was comparable with NOZ cells, and the ectopic expression of the GFP-FLAG protein was validated by Western blot and fluorescence observation (**Figure 2C, D**). L-2F7 cells were sensitive to puromycin with IC50 at 0.77 μg/ml (**Figure 2E**), suggesting that L-2F7 cells are able to accept the most universal gene construction method for puromycin-resistant gene-expressing cell strains in future.

### Morphology and genotype of L-2F7 cells

**Figure 3:**
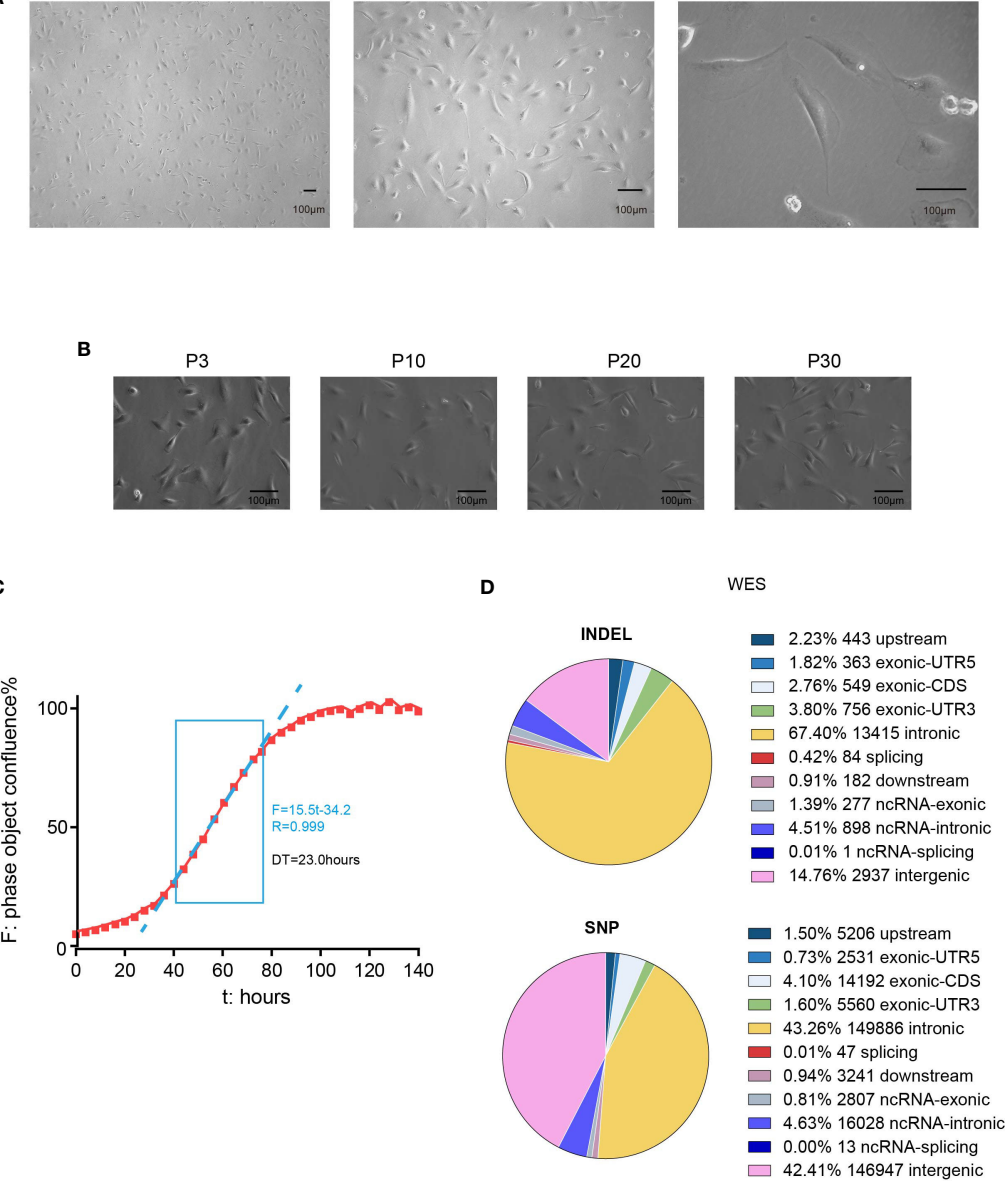
Phenotype and genotype of L-2F7 cells. L-2F7 cellular morphology was shown by microscope images with different magnifications **(A)**. The L-2F7 cellular phenotype remained over 30 passages (P3, P10, P20, P30) **(B)**. The growth curve was plotted with confluence data collected by live cell analysis instrument **(C)**. Whole-exome sequencing including the single-nucleotide polymorphism (SNP) and indel events was shown based on the different classifications of gene candidates **(D)**. DT, doubling time.

L-2F7 cells showed a typical polarity where the nucleus was mainly located at the side of the cell body, instead of central distribution (**Figure 3A**). This cellular phenotype sustained in our long-term culture over 30 passages (**Figure 3B**) and doubling time of L-2F7 for proliferation was 23.0 hours (**Figure 3C**).

We then examined its genomic features through whole-exome sequencing (**Figure 3D**). There were 101 non-synonymous mutations detected including single-nucleotide polymorphism and base insertion and deletion, which were distinct from the hot spots of gene mutations identified in present gallbladder cancer genome studies (**Supplementary Table 1**) (14–17). In addition, neither multi-alleles nor genome cross contamination was detected in the analysis of STR authentication assay (**Table 1**). However, like several other immortalized cell lines including HEK293 (18, 19), the karyotypes of SV40-immortalized cell lines could exhibit polyploids and aneuploids and this phenomenon had already been reported in epithelial tissue-derived cells (20–23). After 30 passages of L-2F7 cells, the karyotype with aneuploids was also found (**Supplementary Figure 2**). Despite of unknown reasons for the chromatin instability at present, L-2F7 cells still exhibited stable cell morphological phenotypes over 30 passages and possessed genomic features distinct from gallbladder cancer and in our study.

**Table 1:** STR authentication in L-2F7 cells.

loci	Allele1	Allele2
D5S818	7	13
D13S317	8	9
D7S820	10	11
D16S539	10	12
VWA	16	17
TH01	7	7
AMEL	X	X
TPOX	8	10
CSF1PO	12	12
D12S391	18	26
FGA	18	23
D2S1338	19	21
D21S11	29	31.2
D18S51	14	22
D8S1179	11	17
D3S1358	16	18
D6S1043	14	19
PENTAE	5	11
D19S433	13	15
PENTAD	9	12
D1S1656	15	17

### Transcriptome pattern of L-2F7 cells

**Figure 4:**
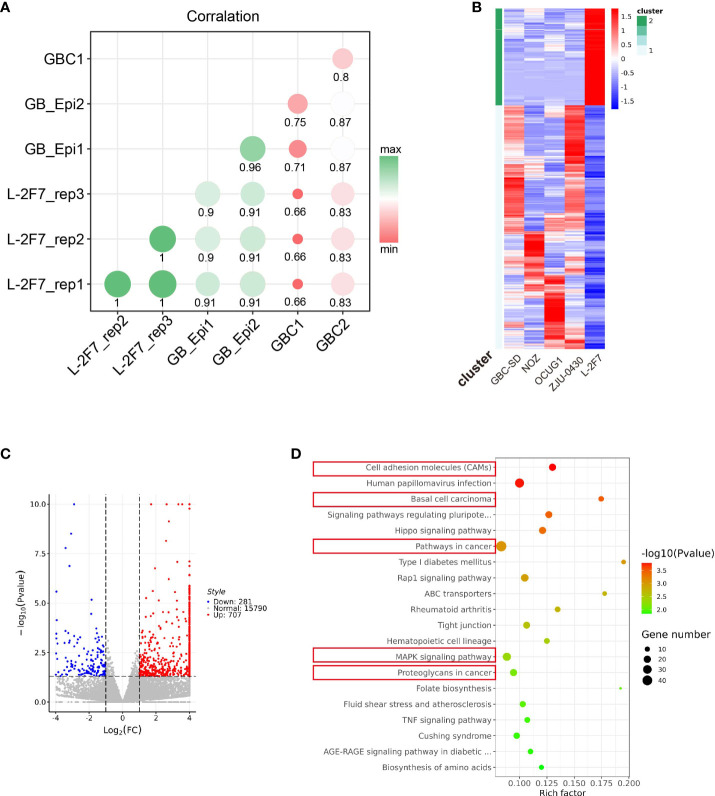
Transcriptome pattern of L-2F7 cells. Transcriptome sequencing was performed in L-2F7 cells, two normal gallbladder epithelium tissues, two gallbladder cancer tissue, and other gallbladder cancer cell lines including NOZ, GBC-SD, ZJU-0430, and OCUG1. The correlation mapping between L-2F7 and various gallbladder tissues was exhibited in the graph **(A)**. Differentially expressed genes were shown in the heatmap **(B)** and volcano plot **(C)**. Gene ontology analysis was performed by comparing L-2F7 and cancer cell lines, and cancer-related pathways were marked with red rectangles **(D)**.

To identify the similarity of this immortalized cell line with its origin tissue features, we compared the transcriptome signature of L-2F7 cells with two datasets individually from normal gallbladder epithelium tissue and gallbladder carcinoma. L-2F7 cells shared a similar differential gene expression to the normal group, but not the two malignant tissues (**Figure 4A**). The differential expression patterns were also validated in the comparison of L-2F7 cells with four other gallbladder cancer cell lines (NOZ, GBC-SD, ZJU-0430, and OCUG1, **Figure 4B**). Of all the candidates detected, 707 genes were upregulated and 281 genes were downregulated in L-2F7 cells (**Figure 4C**). A number of cancer-related pathways were enriched in gallbladder cancer cell lines compared with L-2F7 including basal cell carcinoma and cancer pathways (**Figure 4D**). Moreover, KRAS mutation is one of the most common genomic alternations in gallbladder cancer (15, 24, 25), and KRAS-associated genes were upregulated in GBC cell lines compared with L-2F7 (**Supplementary Figure 3A**). We also observed that estrogen response signaling expressions were elevated in GSEA (**Supplementary Figure 3B, C**), which may account for association with higher incidence of GBC in women than in men (26–28).

### L-2F7 cells acquire motility but fail to develop cancer

**Figure 5:**
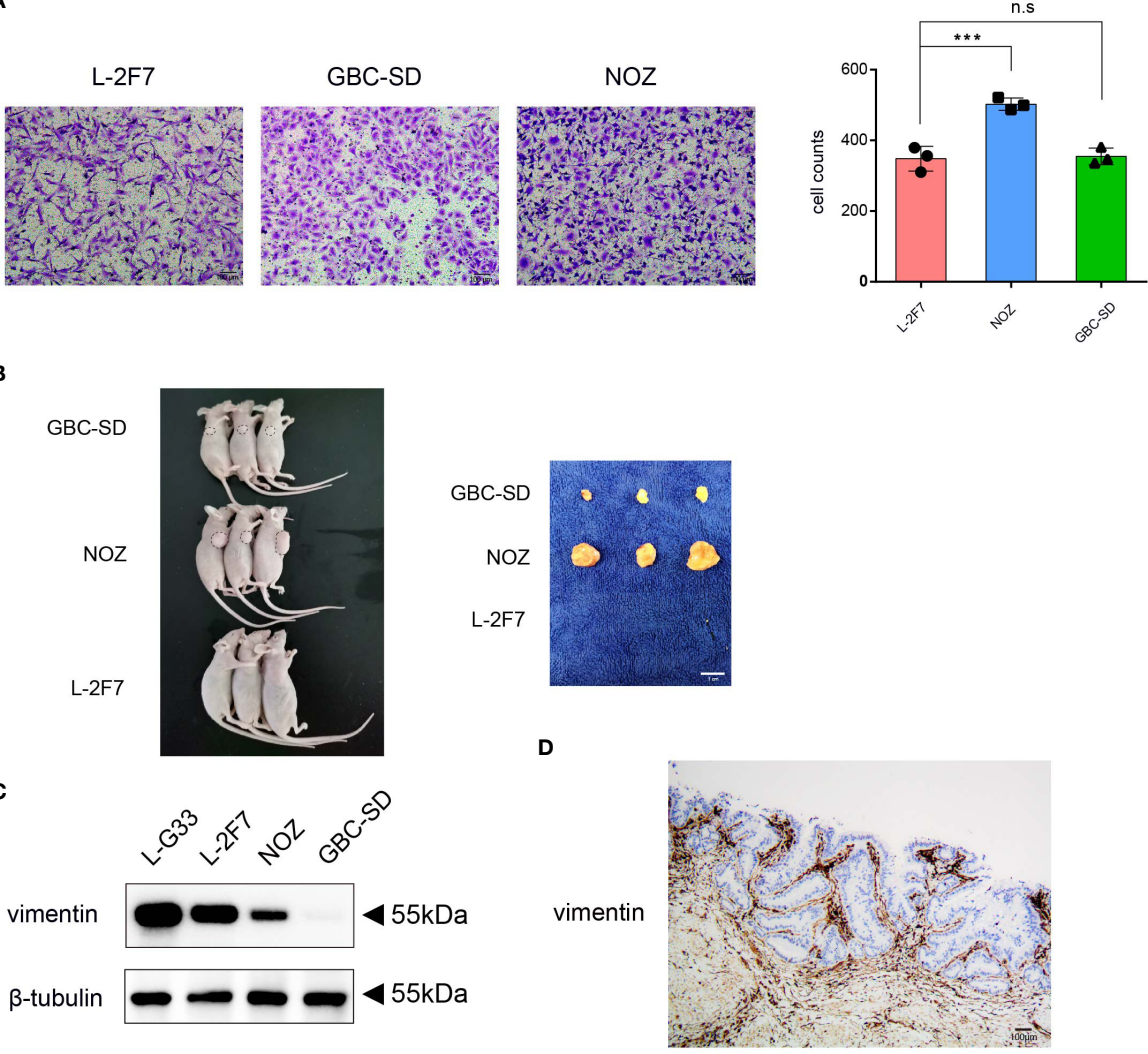
L-2F7 cells acquire motility but fail to develop cancer. L-2F7, NOZ, and GBC-SD cells were tested for their migration ability through Transwell assay, and the migrated cell counts were shown **(A)**. Each cell line (1 × 10^6^) was subcutaneously injected to mice, and 3 weeks later, tumors were removed, n = 3 **(B)**. Expression of vimentin in L-G33, L-2F7, NOZ, and GBC-SD cells was evaluated through Western blot **(C)**. IHC staining of vimentin in normal gallbladder tissue was shown **(D)**. ***p < 0.001; **p < 0.01; *p < 0.05; n.s: non-significant.

To determine if L-2F7 cells possess the ability to migrate as tumor cells, we performed the cell migration assay and found that this cell line possessed an identical ability as GBC-SD cells to migrate (**Figure 5A**). Next, we evaluated if this cell line had the capability of developing tumors *in vivo*; we transplanted L-2F7, NOZ, and GBC-SD cells into nude mice (**Figure 5B**). During the following 3 weeks, we did not find any tumor in the injected site or other organs (i.e., lung, liver) in the L-2F7 group after pathologic necropsy, contrary to other two tumor cell lines that developed local large tumors, indicating that L-2F7 cells are non-tumorigenic. Unexpectedly, we noticed that L-2F7 expressed vimentin comparable with fibroblast L-G33 (**Figure 5C**). Human gallbladder tissue showed a vimentin expression in the duct basal and stromal regions where extensive fibroblasts reside, whereas the epithelium did not express vimentin (**Figure 5D**).

To exclude the possibility of a myoepithelial cell-like subset of the epithelium for L-2F7 cells (29, 30), we analyzed RNA-seq data and found that myoepithelial cell markers S100, KRT4, KRT6, and KRT14 were significantly lower than GBC cells (**Supplementary Figure 4**). The data suggested that the acquitted expression of vimentin by L-2F7 may be partially ascribed to exogenous gene delivery events such as introduction of the SV-40 large T antigen (31, 32). Nonetheless, our data indicate that L-2F7 cells are not tumorigenic and serve as a normal gallbladder epithelial model.

## Discussion

A considerable number of cell lines with physiological states from different organs or tissues have been created and characterized. The availability of these normal cell lines has rendered the research feasible to focus on their physiological function, pathological progression, and cancer transformation (33–35). However, it is unexpectedly noticed that there is lack of a normal gallbladder epithelial cell line, which does emerge as a notable obstacle dampening our interests in gallbladder molecular mechanisms that mediate cellular transformation. Regardless of multiple successful manipulations engaged to establish individual cell lines such as stimulation of normal stem-like cell differentiation and exogenous gene transduction (36), the potential challenges in altered cellular phenotype, function, and/or fate triggered by the enforced genomic alternation should not be neglected. For example, the common genetic transduction with the LT-SV40 gene may lead to other genetic dysregulations, leading to unexpected changes in cellular phenotype and function (32, 37). As a result, these cells undergo genetic transition adaptive to the genome manipulation and environmental stress *in vitro*, the scenario that is distinct from intimate cell–cell interaction *in vivo*. Nevertheless, cell lines created through these genetic manners are at present acceptable and commonly exploited as the normal controls, as these cells in general still sustain the ability to recapitulate biological and physiological signature of their origin *in vivo*.

A number of methodological and technical difficulties in developing cell lines from tissue have raised our great attention. First, fibroblast contamination during tissue isolation appears unavoidable because intense fibroblasts distribute widely just under the epithelium layer and stroma. Second, fibroblasts show the rigorous ability to proliferate rapidly in the cultured plates relative to epithelial cells, where a great variety of growth factors present in culture medium probably meet the full needs of fibroblast metabolism; in contrast, epithelial cells require distinct nutrients unique for their adhesion and growth which are usually deficient or unknown in regular culture. Last, given the nature of terminal differentiation, gallbladder epithelial cells display low division capacity and undergo cell senescence in a short period from tissue isolation. To circumvent these barriers, we first anatomically dissociated the mucosa layer from other tissue layers and subsequently employed the magnetic cell sorting approach using an anti-CD326 antibody to purify epithelial cells. These epithelial cells were then introduced genetically with SV-40 large T antigen and h-TERT for cell immortalization before being individually plated as single clones in 96 multiplate wells.

The whole-exome sequencing showed the unique genotype of L-2F7 with 101 non-synonymous mutations which are divergent from the present hotspot gallbladder cancer genome variation reported. Interestingly, L-2F7 cells have lost the diploidy genome after immortalization and long-term passaging, as this event was identically reported in other epithelial cell lines (23). The substantial mechanisms remain clarified, although it is believed to be associated with the exogenous gene introduction. However, L-2F7 shared a more differential transcriptome profile with the normal gallbladder epithelium than the cancer tissue. In the analysis of differential gene expression profile between L-2F7 cells and four cancer cell lines, 707 genes were found upregulated and 281 genes downregulated in L-2F7 cells, in which the top activated systems involve cell adhesion, basal cell carcinoma, MAPK, and others. Transcriptome analysis suggests that L-2F7 could serve as a normal genome background in the study of GBC tumorigenesis and progression.

It is worthwhile to further investigate if these activated pathways mediate the motility, proliferation, survival, or other functions of L-2F7 cells. For instance, it remains understood if the acquired cell motility resembling cancer activity is associated with the aberrant expression of vimentin.

L-2F7 cells retain the expression of CD326, CK7, CK19, and CK20, but not fibronectin and N-cadherin, underscoring the successful generation of this epithelial cell line. The morphological features maintain as the cells grow in a long-term course of culture (e.g., over 30 passages). Although vimentin was unexpectedly found, which is inconsistent with the absence of vimentin in the gallbladder epithelium, we cannot exclude the possibility that the introduction of LT-SV40 drives the vimentin gene expression as reported in some publications (31, 32, 37). In addition, L-2F7 cells are not myoepithelial-like cells because they are deficient in myoepithelial cell markers such as S100, KRT4, KRT6, and KRT14.

To the end, we have validated that L-2F7 cells are non-tumorigenic, in contrast with epithelium-derived cancer lines GBC-SD and NOZ that developed palpable tumors in xenografted models. Collectively, we have established a non-cancerous gallbladder epithelial cell line L-2F7 that could serve as valuable normal epithelial cells for the study in gallbladder carcinogenesis and other gallbladder-related diseases.

## Methods

### Primary cell extraction

A part of the gallbladder tissue was cut and stored on ice in a tissue storage solution (130-100-008, Miltenyi Biotech (Bergisch Gladbach, North Rhine-Westphalia, Germany)). The lumenal mucus layers of the gallbladder tissue were isolated anatomically by scrapes then digested with 5 ml of a tissue dissociation buffer (130-095-929, Miltenyi Biotech) in a 37°C shaker for approximately 20 min. A cell medium with 5 ml of RPMI: Roswell Park Memorial Institute (SH30809.01, Cyvita (Logan, Utah, USA)) containing 10% Fetal Bovine Serum (FBS) (164210, Procell (Wuhan, Hubei Province, China)) was added prior to filtering with a 40-µm strainer (352340, Falcon (One Becton, Durham, USA)). After centrifugation for 1,000×g, 5 min, the cells were suspended in 1 ml of a red cell lysis buffer (W0126, Tiangen (Beijing, China)) on ice for 3 min and finally, washed with the complete medium two times.

### Magnetic cell sorting

The above collected cells were subjected to the removal of dead cells using a dead-cell removal kit (130-090-101, Miltenyi Biotech), then CD326 positive cells were sorted following the instruction in the CD326 microbeads human kit (130-061-101, Miltenyi Biotech).

### Cell culture

All the primary cells in this study were cultured in EpiCM (4101, Sciencell (San Diego, California, USA)), but we modified the kit instruction with the replacement of 2% FBS instead of 10% FBS to culture all single cell clones including the epithelial clone L-2F7 and fibroblast clone L-G33. As immortalized gene vectors harbored antibiotic resistance genes, hygromycin (60224ES03, Yeasen) and blasticidin (60218ES10, Yeasen (Shanghai, China)) were selected at concentrations of 200 and 10µg/ml, respectively. The gallbladder cancer cell (GBC) lines NOZ, GBC-SD, ZJU-0430, and OCUG1 were authenticated by Short tandem (STR) repeat and tested if mycoplasma-free. The four GBC cell lines were cultured in Dulbecco's Modified Eagle Medium (DMEM) (SH30243.01, Hyclone (Logan, Utah, USA)) with 10% FBS and 1% penicillin–streptomycin solution (60162ES76, Yeasen (Shanghai, China)). All the cells were digested by a trypsin solution (40127ES60, Yeasen) before passaging or being frozen with a cell-saving buffer (C30100, NCM (Suzhou, Jiangsu Province, China) Biotech).

### Cell immortalization

LT-SV40 was subcloned into CV301 vector (Genechem (Shanghai, China)) with blasticidin resistance gene and hTERT was subcloned into CV237 vector (Genechem (Shanghai, China)) with hygromycin resistance gene. Lentivirus was developed by co-transfection into 293T with pHelper 1.0 (Genechem), pHelper 2.0 (Genechem (Shanghai, China)), and the above two vectors. Then, the concentrated viral medium was added into a cell culture medium with 10 µg/ml polybrene (40804ES76, Yeasen). Twenty-four hours later, the culture medium was replaced with the modified EpiCM medium in the presence of blasticidin and hygromycin

### Flow cytometry assay

Cells were collected after digestion by trypsin, washed with Phosphate Buffered Saline for two times and were incubated with a Phycoerythrin (PE)-conjugated anti-human CD326 antibody (324205, Biolegend (San Diego, California, USA)) for 1 h at 4°C. Samples were examined by BD FACS Celesta cell analyzer (BD bioscience, Franklin Lakes, New Jersey, USA) and FACS data were processed by FlowJo_v10 software.

### Cell lysis, antibodies, and Western blotting

After being washed with Phosphate Buffered Saline (PBS), cells were lysed by a protein lysis buffer (P0013, Beyotime) containing 1 mM of Phenylmethanesulfonyl fluoride (PMSF) (ST506, Beyotime (Shanghai, China)). Protein lysates were quantified by Bradford assay (1863028, Thermo Fisher Scientific (Waltham, Massachusetts, USA)). Western blotting was carried out according to standard methods. Antibodies specific to fibronectin (1561301-AP, Proteintech (Wuhan, Hubei Province, China)), CK19 (10712-AP, Proteintech), CK7 (15539-1, Proteintech), CK20 (17329-1-AP, Proteintech), N-cadherin (13116S, Cell Signaling Technology), β-tubulin (T8328, Sigma-Aldrich), MRP3 (39909S, Cell Signaling Technology (Danvers, Massachusetts, USA)), Flag tag (F1804, Signal-Aldrich (Burlington, Massachusetts, USA)), GFP tag (AED011, ABclonal (Wuhan, Hubei Province, China)),Vimentin (A19607, ABclonal), LT-SV40 (sc-147, Santacruz (Dallas, Texas, USA)), hTERT (17329-1-AP, Proteintech), TP53 (2527S, CST), and Rb (9313S, CST) were applied for Western blotting.

### Immunofluorescence 

Cells were fixed in precooled (-20°C) methyl alcohol for 5 min and incubated with 0.1% Triton-100 for 10 min. Samples were then incubated in diluted primary antibodies at 4°C overnight followed by a secondary antibody conjugated with Alexa Fluorescence 488 (33116ES60, Yeasen) in 37°C for 1 h. Nuclei were stained with DAPI (P0131, Beyotime), and finally, the samples were mounted.

### Cell growth curves

Cells were passaged to individual wells of 96-well plates at a density of 10%, then live cells were analyzed with a live-cell analysis instrument (Incucyte S3, Sartorius (Göttingen, Germany)). Cell culture images were saved every 4 h and the cell confluence rates were calculated with built-in software according to the instrument brochure. The raw data were fitted to an exponential model with logarithmic phases.

### Xenografted models *in vivo*

Nude mice (nu/nu, 6- to 8-week-old males) were injected subcutaneously with 1 × 106 cells. After 3 weeks, the mice were sacrificed and tumors were dissected and measured. Animal-related procedures were performed after the approval of the Institutional Animal Care and Use Committee of the Renji Hospital affiliated to Shanghai Jiao Tong University School of Medicine.

### Whole-exome sequencing

Cellular genomic DNA was extracted and constructed to a genomic library with NEB Next® Ultra™ DNA Library Prep Kit for Illumina (NEB, Ipswich, Massachusetts, USA), and then sequenced in Illumina (San Diego, California, USA) HiSeq X10. The BWA-MEM software mem software was used to align the sequence with the reference genome UCSC hg19. Finally, GATK was used to detect variations that were annotated by the ANNOVAR software.

### Bulk RNA sequencing

RNA was extracted with TRIZOL (15596018, Thermo Fisher) and constructed to a library with NEB Next Ultra™ RNA Library Prep Kit (NEB, USA), and then sequenced in Novaseq 6000, PE150. The Trimmomatic software was used to remove the adapter and low-quality reads. The STAR software was used to align clean reads to the reference genome. Finally, analysis was performed using the Gene Ontology (GO) analysis, GSEA software (Broad Institute, Cambridge, MA, USA), and the Kyoto Encyclopedia of Genes and Genomes (KEGG) database. Correlation analysis was completed with the cor() function.

### Cell migration assay

Twenty thousand cells were suspended in 200-µl FBS-free DMEM medium and then placed in the upper chamber of the transwell insert (3422, Corning (Corning, New York, USA)). Then 600 µl of DMEM with 10% FBS was added in the lower chamber. After 24 h, the inserts were collected and fixed in a 10% formaldehyde solution for 5 min before being stained in a 0.5% crystal violet stain solution (60506ES60, Yeasen) for 5 min. Cells on the upper chamber of the insert were removed carefully by swabs and the number of migrated cells were pictured and counted under the microscope.

### Short tandem repeat authentication

DNA was extracted by a DNA extraction kit (AP-EMN-BL-GDNA-250, Corning). Twenty STRs including the Amelogenin locus were amplified by six multiplex PCR and separated on an ABI 3730XL Genetic Analyzer. The signals were then analyzed by the software GeneMapper.

### Karyotyping

The L-2F7 passaged over 30 times was used to analyze the karyotypes. When cell density was 60–70%, cells were prevented in metaphase by exposing to 0.2 µg/ml of demecolcine for 3 h followed by suspension in 0.8% sodium citrate for 30 min. Cells were fixed with 4 ml of acetic methanol solution for 20 min and then dropped on slides before placement at 75°C for 3 h. Finally, a 10% Giemsa solution was used for staining prior to counting the chromosomes. Twenty slides were analyzed.

### Statistical analysis

GraphPad Prism v4.0 was used to create plots and applied for data analysis. Student’s t-test was used. P-values <0.05 were considered statistically significant. All experiments were repeated for more than three times.

## Data availability statement

This work was supported by National Natural Science Foundation of China (No. 32130036).

## Ethics statement

This study was reviewed and approved by Shanghai Jiaotong University School of Medicine, Renji Hospital Ethics Committee. The patients/participants provided their written informed consent to participate in this study. The animal study was reviewed and approved by the Institutional Animal Care and Use Committee of Renji Hospital affiliated to Shanghai Jiao Tong University School of Medicine.

## Author contributions

ZW, SW, and ZJ performed main experiments and drafted the manuscript. TC, WY collected clinical samples. YZ and MY coordinated and edited the drafting of the manuscript. RS, DX and YL revised and edited the final version of the manuscript. All authors contributed to the article and approved the submitted version.

## Funding

This work was supported by National Natural Science Foundation of China (No. 32130036).

## Conflict of interest

The authors declare that the research was conducted in the absence of any commercial or financial relationships that could be construed as a potential conflict of interest.

## Publisher’s note

All claims expressed in this article are solely those of the authors and do not necessarily represent those of their affiliated organizations, or those of the publisher, the editors and the reviewers. Any product that may be evaluated in this article, or claim that may be made by its manufacturer, is not guaranteed or endorsed by the publisher.

## Supplementary material

Supplementary Figure 1 The SV40 and hTERT expression were tested in L-2F7 compared with gallbladder epithelium tissue lysis **(A)**. TP53 and Rb expression were compared with NOZ and ZJU0430 cells in WB **(B)**. The epithelium markers including CK7 and CK19 at different passaging stages in L-2F7 were shown **(C)**. The expression of CK19 **(D)** and fibronectin **(E)** was examined through immunofluorescence in L-2F7, NOZ, GBC-SD and L-G33 cells.

Supplementary Figure 2 Karyotyping of L-2F7 cells was performed and L-2F7 cells exhibited aneuploids.

Supplementary Figure 3 GSEA analysis were performed to compare GBC cell lines with L-2F7 cells, and the pathways on KRAS **(A)** and estrogen related signals **(C, D)** were shown.

Supplementary Figure 4 Myoepithelial markers (S100, KRT4, KRT6, KRT14) were analyzed through RNAseq data of L-2F7 cell and other four cancer cell lines, and the expression levels were shown in heatmap.

Supplementary Table 1 genomic alternations of L-2F7. The whole exome sequencing analysis of L-2F7, single nucleotide polymorphism, base insertion and deletions in genes were shown in the table.

## References

[1] Gilloteaux J. Introduction to the biliary tract, the gallbladder, and gallstones. Microscopy Res Technique (1997) 38(6):547–51. doi: 10.1002/(SICI)1097-0029(19970915)38:6<547::AID-JEMT1>3.0.CO;2-C 9330345

[2] Abiru H Sarna SK Condon RE. Contractile mechanisms of gallbladder filling and emptying in dogs. Gastroenterology (1994) 106(6):1652–61. doi: 10.1016/0016-5085(94)90423-5 8194713

[3] Tierney S Pitt HA Lillemoe KD. Physiology and pathophysiology of gallbladder motility. Surg Clinics North America (1993) 73(6):1267–90. doi: 10.1016/S0039-6109(16)46191-8 8248838

[4] Housset C Chretien Y Debray D Chignard N. Functions of the gallbladder. Compr Physiol (2016) 6(3):1549–77. doi: 10.1002/cphy.c150050 27347902

[5] Frierson HF Jr. The gross anatomy and histology of the gallbladder, extrahepatic bile ducts, vaterian system, and minor papilla. Am J Surg Pathol (1989) 13(2):146–62. doi: 10.1097/00000478-198902000-00008 2644853

[6] Oldham-Ott CK Gilloteaux J. Comparative morphology of the gallbladder and biliary tract in vertebrates: variation in structure, homology in function and gallstones. Microsc Res Tech (1997) 38(6):571–97. doi: 10.1002/(SICI)1097-0029(19970915)38:6<571::AID-JEMT3>3.0.CO;2-I 9330347

[7] Hundal R Shaffer EA. Gallbladder cancer: epidemiology and outcome. Clin Epidemiol (2014) 6:99–109. doi: 10.2147/CLEP.S37357 24634588 PMC3952897

[8] Liu Yingbin CW. Attach importance to the standardized diagnosis and treatment of gallbladder carcinoma. Zhonghua Wai Ke Xue Za Zhi (2021) 59(4):249–54. doi: 10.3760/cma.j.cn112139-20210115-00031 33706440

[9] Obara T Tanno S Mizukami Y Fuji T Izawa T Yanagawa N. Epithelial cell proliferation and gene mutation in the mucosa of gallbladder with pancreaticobiliary malunion and cancer. J Hepatobil Pancreat Surg (1999) 6(3):229–36. doi: 10.1007/s005340050112 10526057

[10] Feng F Cheng Q Li B Liu C Wang H Li B. Establishment and characterization of 38 novel patient-derived primary cancer cell lines using multi-region sampling revealing intra-tumor heterogeneity of gallbladder carcinoma. Hum Cell (2021) 34(3):918–31. doi: 10.1007/s13577-021-00492-5 PMC8057967 33813726

[11] Zhou F Zhang Y Sun J Yang X. Characteristics of a novel cell line ZJU-0430 established from human gallbladder carcinoma. Cancer Cell Int (2019) 19:190. doi: 10.1186/s12935-019-0911-1 31367188 PMC6647153

[12] Trauner M Boyer JL. Bile salt transporters: molecular characterization, function, and regulation. Physiol Rev (2003) 83(2):633–71. doi: 10.1152/physrev.00027.2002 12663868

[13] Rost D Konig J Weiss G Klar E Stremmel W Keppler D. Expression and localization of the multidrug resistance proteins MRP2 and MRP3 in human gallbladder epithelia. Gastroenterology (2001) 121(5):1203–8. doi: 10.1053/gast.2001.28648 11677213

[14] Li M Liu F Zhang F Zhou W Jiang X Yang Y. Genomic ERBB2/ERBB3 mutations promote PD-L1-mediated immune escape in gallbladder cancer: A whole-exome sequencing analysis. Gut (2018) 68(6):1024–33. doi: 10.1136/gutjnl-2018-316039 29954840

[15] Li M Zhang Z Li X Ye J Wu X Tan Z. Whole-exome and targeted gene sequencing of gallbladder carcinoma identifies recurrent mutations in the ErbB pathway. Nat Genet (2014) 46(8):872–6. doi: 10.1038/ng.3030 24997986

[16] Kuipers H de Bitter TJJ de Boer MT van der Post RS Nijkamp MW de Reuver PR. Gallbladder cancer: Current insights in genetic alterations and their possible therapeutic implications. Cancers (Basel) (2021) 13(21):5257. doi: 10.3390/cancers13215257 34771420 PMC8582530

[17] Mishra S Kumari S Srivastava P Pandey A Shukla S Husain N. Genomic profiling of gallbladder carcinoma: Targetable mutations and pathways involved. Pathol Res Pract (2022) 232:153806. doi: 10.1016/j.prp.2022.153806 35231860

[18] Bylund L Kytola S Lui WO Larsson C Weber G. Analysis of the cytogenetic stability of the human embryonal kidney cell line 293 by cytogenetic and STR profiling approaches. Cytogenet Genome Res (2004) 106(1):28–32. doi: 10.1159/000078556 15218237

[19] Lin YC Boone M Meuris L Lemmens I Van Roy N Soete A. Genome dynamics of the human embryonic kidney 293 lineage in response to cell biology manipulations. Nat Commun (2014) 5:4767. doi: 10.1038/ncomms5767 25182477 PMC4166678

[20] Akagi T Sasai K Hanafusa H. Refractory nature of normal human diploid fibroblasts with respect to oncogene-mediated transformation. Proc Natl Acad Sci U.S.A. (2003) 100(23):13567–72. doi: 10.1073/pnas.1834876100 PMC263854 14597713

[21] Bernal A Zafon E Domínguez D Bertran E Tusell L. Generation of immortalised but unstable cells after hTERT introduction in telomere-compromised and p53-deficient vHMECs. Int J Mol Sci (2018) 19(7):2078. doi: 10.3390/ijms19072078 PMC6073565 30018248

[22] Neufeld DS Ripley S Henderson A Ozer HL. Immortalization of human fibroblasts transformed by origin-defective simian virus 40. Mol Cell Biol (1987) 7(8):2794–802. doi: 10.1128/mcb.7.8.2794-2802.1987 PMC367896 2823105

[23] Velicescu M Yu J Herbert BS Shay JW Granada E Dubeau L. Aneuploidy and telomere attrition are independent determinants of crisis in SV40-transformed epithelial cells. Cancer Res (2003) 63(18):5813–20. 14522904

[24] Kuroki T Tajima Y Matsuo K Kanematsu T. Genetic alterations in gallbladder carcinoma. Surg Today (2005) 35(2):101–5. doi: 10.1007/s00595-004-2906-2 15674488

[25] Iyer P Shrikhande SV Ranjan M Joshi A Gardi N Prasad R. ERBB2 and KRAS alterations mediate response to EGFR inhibitors in early stage gallbladder cancer. Int J Cancer (2019) 144(8):2008–19. doi: 10.1002/ijc.31916 PMC6378102 30304546

[26] Gabbi C Kim HJ Barros R Korach-Andre M Warner M Gustafsson JA. Estrogen-dependent gallbladder carcinogenesis in LXRbeta-/- female mice. Proc Natl Acad Sci U.S.A. (2010) 107(33):14763–8. doi: 10.1073/pnas.1009483107 PMC2930435 20679224

[27] Gupta P Agarwal A Gupta V Singh PK Pantola C Amit S. Expression and clinicopathological significance of estrogen and progesterone receptors in gallbladder cancer. Gastrointest Cancer Res (2012) 5(2):41–7. PMC3369597 22690257

[28] Hryciuk B Peksa R Bienkowski M Szymanowski B Radecka B Winnik K. Expression of female sex hormone receptors, connective tissue growth factor and HER2 in gallbladder cancer. Sci Rep (2020) 10(1):1871. doi: 10.1038/s41598-020-58777-y 32024900 PMC7002405

[29] Balachander N Masthan KM Babu NA Anbazhagan V. Myoepithelial cells in pathology. J Pharm Bioallied Sci (2015) 7(Suppl 1):S190–3. doi: 10.4103/0975-7406.155898 PMC4439666 26015706

[30] Deugnier MA Teuliere J Faraldo MM Thiery JP Glukhova MA. The importance of being a myoepithelial cell. Breast Cancer Res (2002) 4(6):224–30. doi: 10.1186/bcr459 PMC137933 12473168

[31] Rathje LS Nordgren N Pettersson T Ronnlund D Widengren J Aspenstrom P. Oncogenes induce a vimentin filament collapse mediated by HDAC6 that is linked to cell stiffness. Proc Natl Acad Sci U.S.A. (2014) 111(4):1515–20. doi: 10.1073/pnas.1300238111 PMC3910606 24474778

[32] Ahuja D Saenz-Robles MT Pipas JM. SV40 large T antigen targets multiple cellular pathways to elicit cellular transformation. Oncogene (2005) 24(52):7729–45. doi: 10.1038/sj.onc.1209046 16299533

[33] Ramboer E Vanhaecke T Rogiers V Vinken M. Immortalized human hepatic cell lines for in vitro testing and research purposes. Methods Mol Biol 2015 (2015) 1250:53–76. doi: 10.1007/978-1-4939-2074-7_4 PMC4579543 26272134

[34] Okayama N Fowler MR Jennings SR Patel P Specian R Alexander B. Characterization of jok-1, a human gastric epithelial cell line. in vitro. Cell Dev Biol - Anim (2000) 36(4):228–34. doi: 10.1290/1071-2690(2000)036<0228:COJAHG>2.0.CO;2 10852347

[35] Ouyang H Mou Luk L-j C Liu N Karaskova J Squire J. Immortal human pancreatic duct epithelial cell lines with near normal genotype and phenotype. Am J Pathol (2000) 157(5):1623–31. doi: 10.1016/S0002-9440(10)64800-6 PMC1885733 11073822

[36] Gudjonsson T Villadsen R Ronnov-Jessen L Petersen OW. Immortalization protocols used in cell culture models of human breast morphogenesis. Cell Mol Life Sci (2004) 61(19-20):2523–34. doi: 10.1007/s00018-004-4167-z PMC11924520 15526159

[37] Rotondo JC Mazzoni E Bononi I Tognon M Martini F. Association between simian virus 40 and human tumors. Front Oncol (2019) 9:670. doi: 10.3389/fonc.2019.00670 31403031 PMC6669359

