# Supplementary figures and images for: Establishment and characterization of an immortalized epithelial cell line from human gallbladder
Source: Front Oncol. 2022 Oct 28;12:994087. doi: 10.3389/fonc.2022.994087 (PMC9650220; doi:10.3389/fonc.2022.994087)

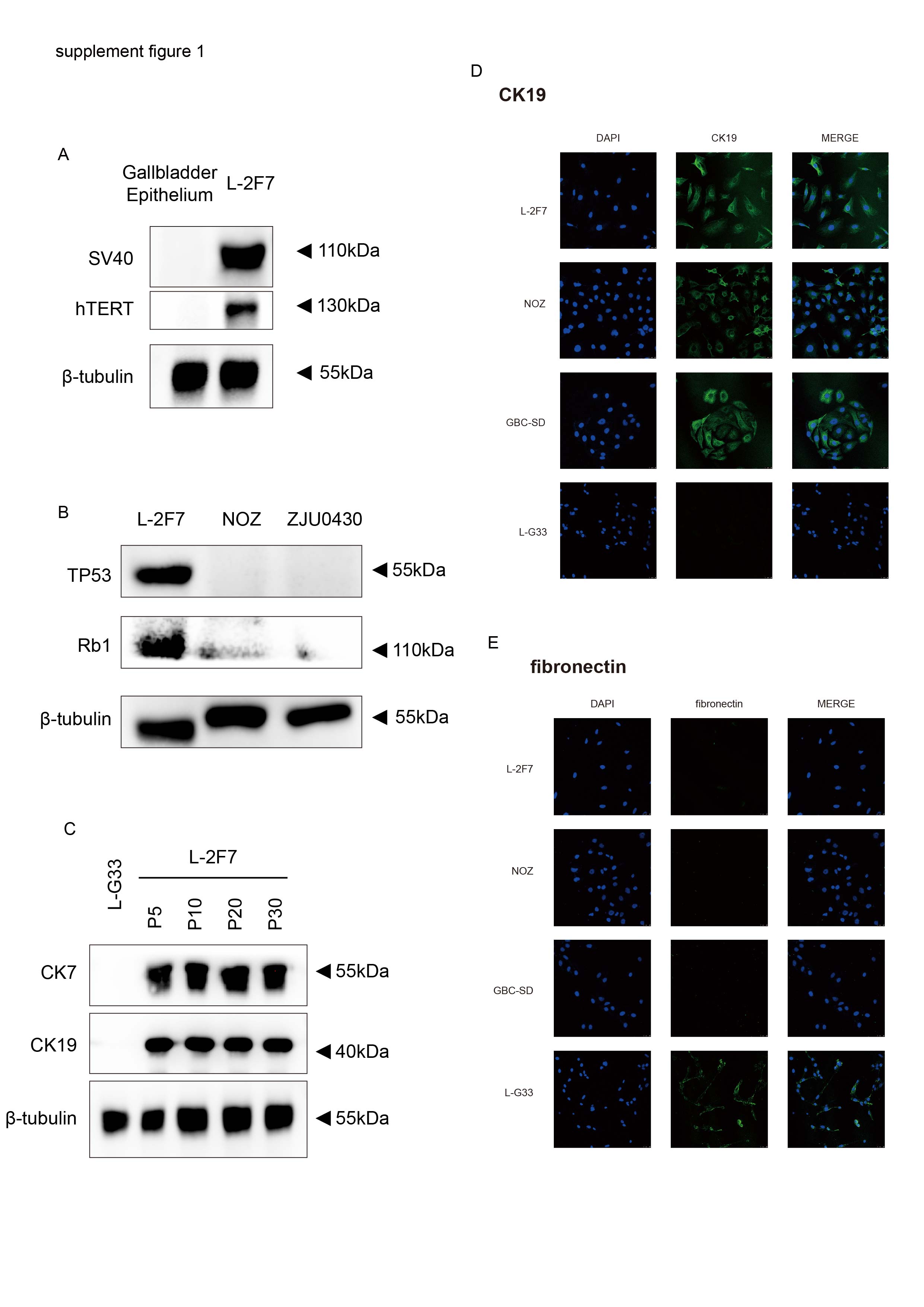

Supplement: Supplementary Figure 1 — The SV40 and hTERT expression were tested in L-2F7 compared with gallbladder epithelium tissue lysis (A). TP53 and Rb expression were compared with NOZ and ZJU0430 cells in WB (B). The epithelium markers including CK7 and CK19 at different passaging stages in L-2F7 were shown (C). The expression of CK19 (D) and fibronectin (E) was examined through immunofluorescence in L-2F7, NOZ, GBC-SD and L-G33 cells. [file Image_1.jpeg]

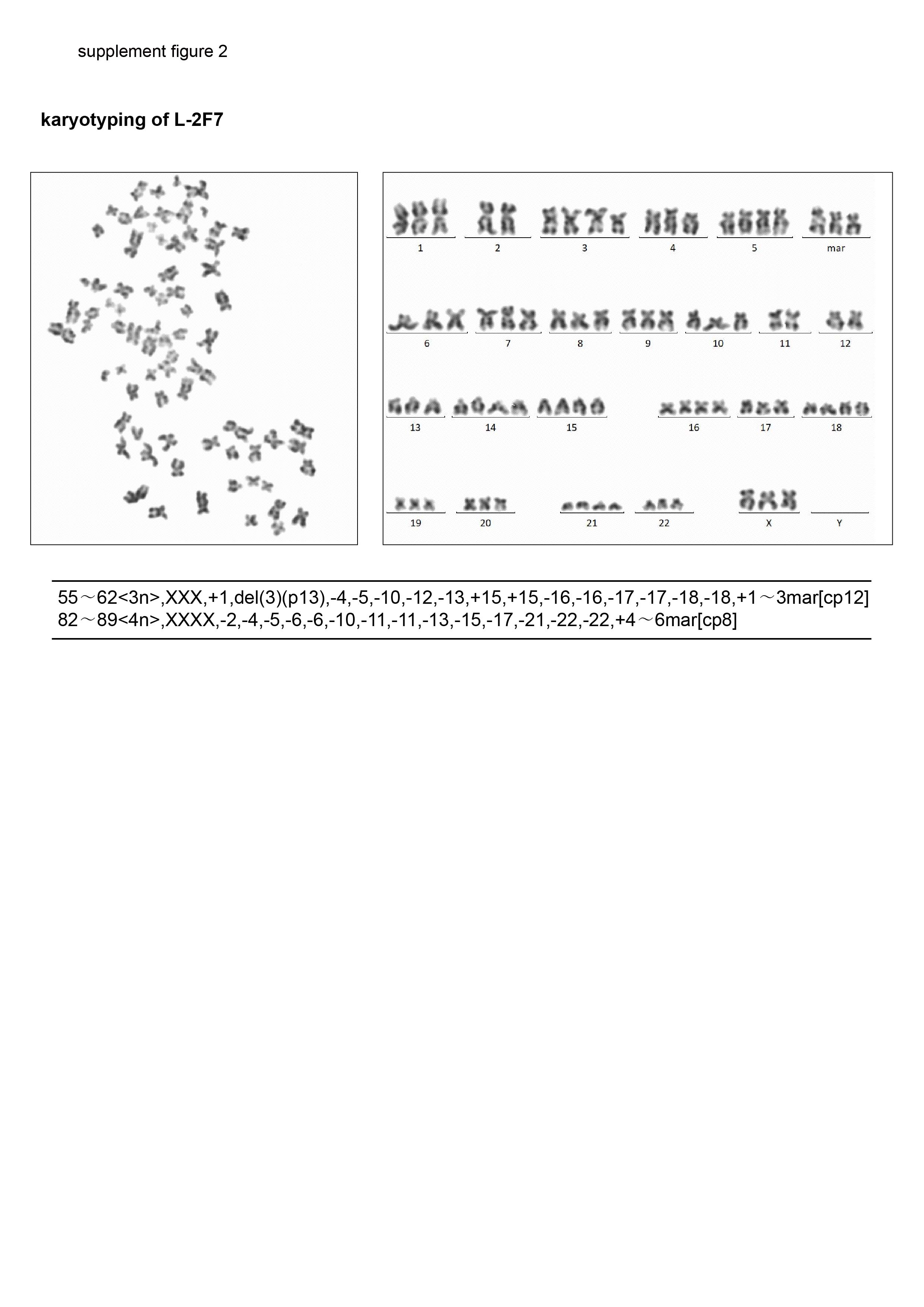

Supplement: Supplementary Figure 2 — Karyotyping of L-2F7 cells was performed and L-2F7 cells exhibited aneuploids. [file Image_2.jpeg]

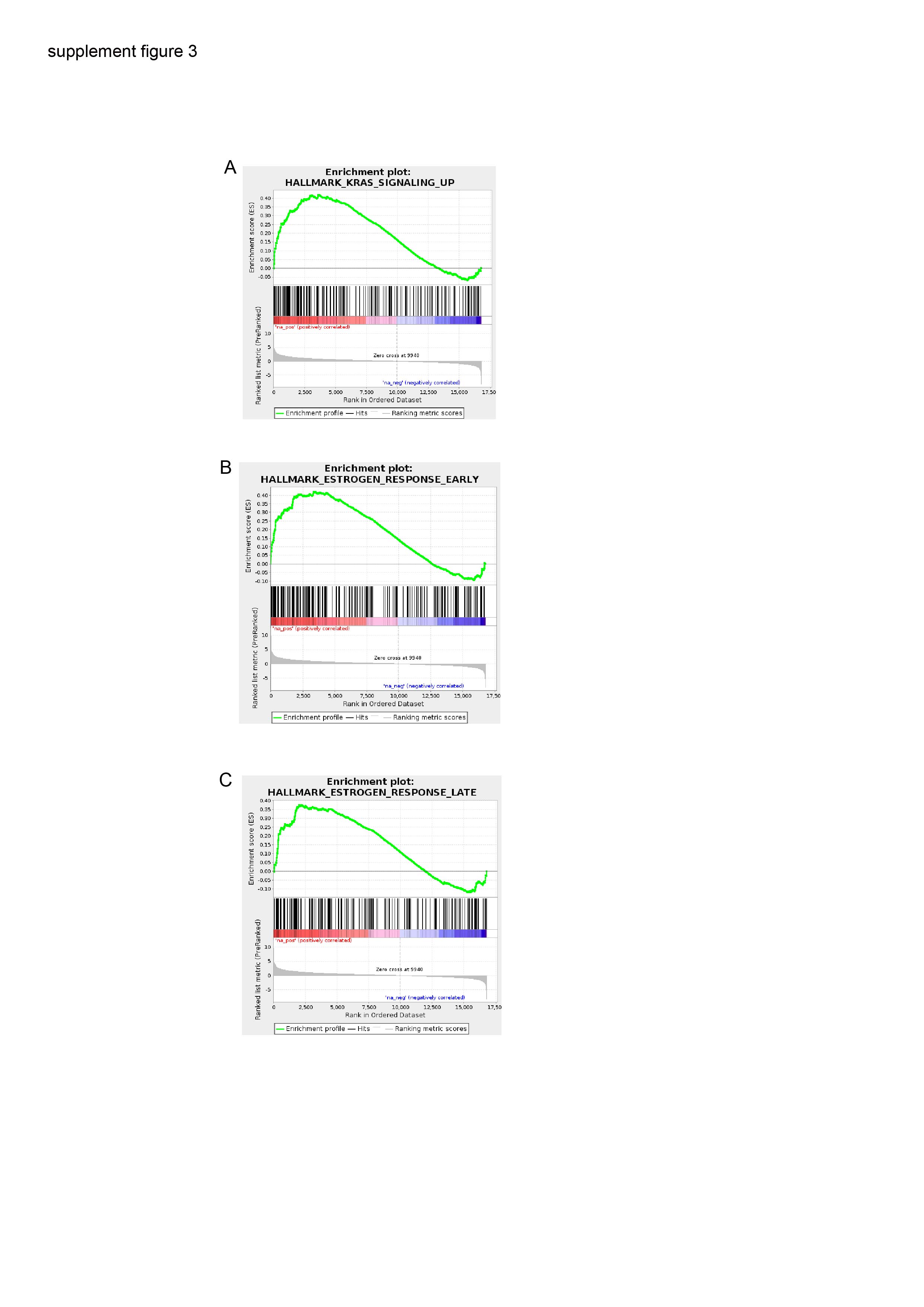

Supplement: Supplementary Figure 3 — GSEA analysis were performed to compare GBC cell lines with L-2F7 cells, and the pathways on KRAS (A) and estrogen related signals (C, D) were shown. [file Image_3.jpeg]

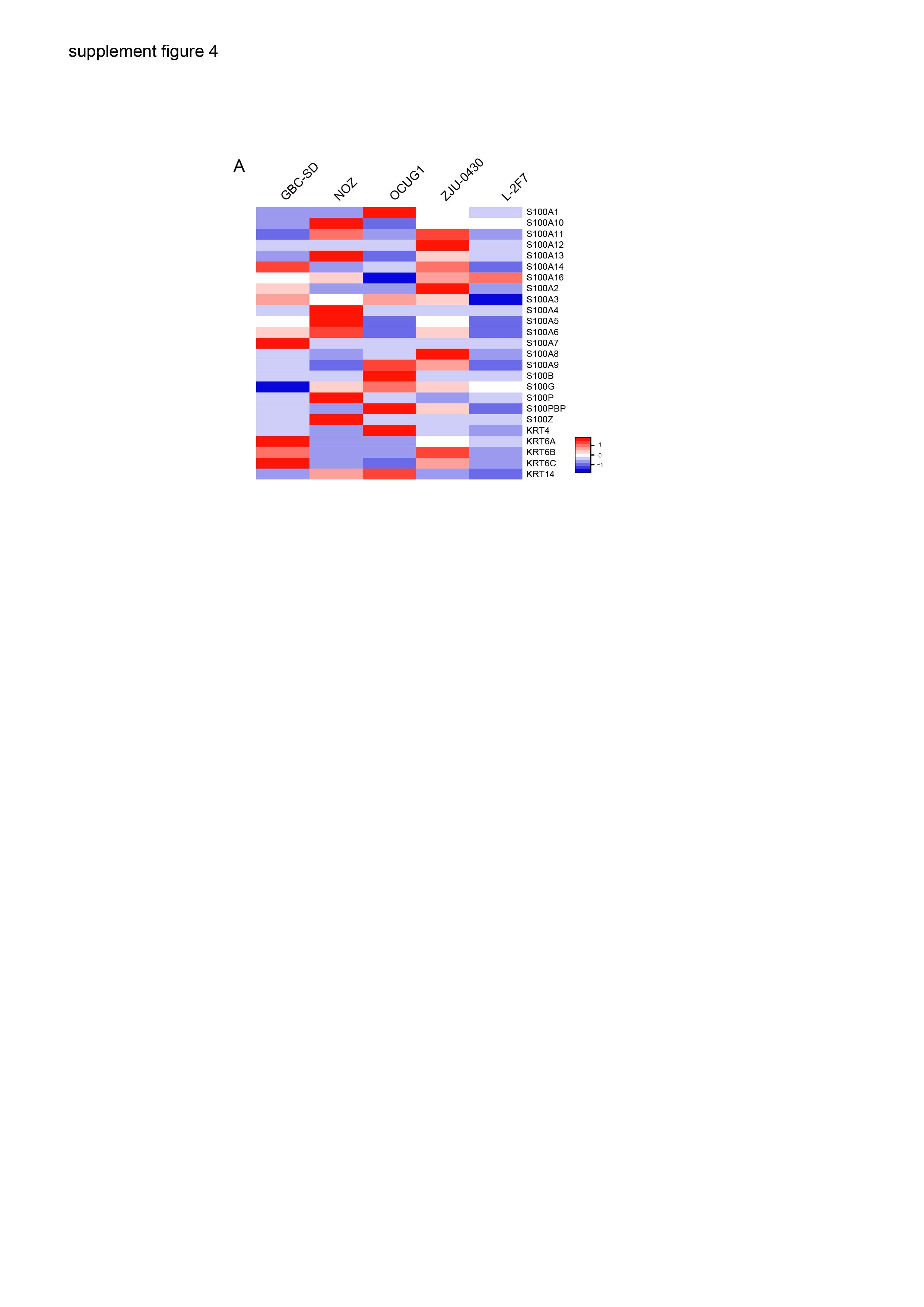

Supplement: Supplementary Figure 4 — Myoepithelial markers (S100, KRT4, KRT6, KRT14) were analyzed through RNAseq data of L-2F7 cell and other four cancer cell lines, and the expression levels were shown in heatmap. [file Image_4.jpeg]
